# Comparative Proteomics Analysis of Placenta from Pregnant Women with Intrahepatic Cholestasis of Pregnancy

**DOI:** 10.1371/journal.pone.0083281

**Published:** 2013-12-31

**Authors:** Ting Zhang, Yueshuai Guo, Xuejiang Guo, Tao Zhou, Daozhen Chen, Jingying Xiang, Zuomin Zhou

**Affiliations:** 1 State Key Laboratory of Reproductive Medicine, Department of Histology and Embryology, Nanjing Medical University, Nanjing, China; 2 Wuxi Maternity and Child Health Care Hospital Affiliated to Nanjing Medical University, Wuxi, China; Institute of Zoology, Chinese Academy of Sciences, China

## Abstract

**Introduction:**

Intrahepatic cholestasis of pregnancy (ICP) usually occurs in the third trimester and associated with increased risks in fetal complications. Currently, the exact cause of this disease is unknown. In this study we aim to investigate the potential proteins in placenta, which may participate in the molecular mechanisms of ICP-related fetal complications using iTRAQ-based proteomics approach.

**Methods:**

The iTRAQ analysis combined with liquid chromatography-tandem mass spectrometry (LC-MS/MS) was performed to separate differentially expressed placental proteins from 4 pregnant women with ICP and 4 healthy pregnant women. Bioinformatics analysis was used to find the relative processes that these differentially expressed proteins were involved in. Three apoptosis related proteins ERp29, PRDX6 and MPO that resulted from iTRAQ-based proteomics were further verified in placenta by Western blotting and immunohistochemistry. Placental apoptosis was also detected by TUNEL assay.

**Results:**

Proteomics results showed there were 38 differentially expressed proteins from pregnant women with ICP and healthy pregnant women, 29 were upregulated and 9 were downregulated in placenta from pregnant women with ICP. Bioinformatics analysis showed most of the identified proteins was functionally related to specific cell processes, including apoptosis, oxidative stress, lipid metabolism. The expression levels of ERp29, PRDX6 and MPO were consistent with the proteomics data. The apoptosis index in placenta from ICP patients was significantly increased.

**Conclusion:**

This preliminary work provides a better understanding of the proteomic alterations of placenta from pregnant women with ICP and may provide us some new insights into the pathophysiology and potential novel treatment targets for ICP.

## Introduction

Intrahepatic cholestasis of pregnancy (ICP) is the specific pregnancy-related liver disease which occurs at the third trimester of pregnancy. ICP is characterized by pruritus and elevated liver enzymes and/or serum bile acids [1], [2], and the disease symptoms and liver dysfunction resolve quickly after delivery. However, ICP can lead to complications for both mother and fetus, and is associated with an increased risk of spontaneous preterm labor, fetal distress and sudden intrauterine death [3]–[6]. Currently, the exact cause of this disease is unknown. The risk of adverse fetal outcomes is reportedly increased in pregnancies when the maternal bile acid levels exceed 40 µmol/L [6]. Therefore, bile acids are likely to play a key role in the pathogenesis of ICP.

Under physiological circumstances, the placenta plays a crucial role in protecting the fetus from the adverse effects of potentially toxic endogenous substances, including bile acids [7], or xenobiotics that reach the maternal circulation [8]. Disturbances to placental function may challenge this protection. Moreover, a role for the placenta in the development of ICP has been suggested, due to the disappearance of pruritus and the normalization of liver function tests after delivery of the placenta [9], [10]. Studies have also shown that high concentrations of bile acids can induce apoptosis in the placenta, and the incidence of apoptosis decreases after treatment with ursodeoxycholic acid (UDCA), which protects the placenta from the toxic effect of bile acids and commonly used in the management of ICP [9], [11]. All these findings suggest that a high concentration of bile acids may play a key role in damage to the placenta, and also participate in the molecular pathogenesis of ICP and the incidence of adverse fetal outcomes.

However, the mechanisms responsible for placental apoptosis in ICP patients have not yet been clearly identified. Moreover, it is not clear whether other pathological changes occur in the placenta from ICP patients with a high concentration of bile acids. Identification of the placental proteins which are affected in ICP is indispensable to our understanding of the complex molecular background associated with this multifactorial event. To investigate the changes in the proteome of placenta from pregnant women with ICP, an isobaric tags for relative and absolute quantification (iTRAQ) - based proteomics approach was performed in this study. The identification of differentially expressed proteins may help to facilitate a better understanding of the molecular mechanisms of ICP-related fetal complications.

## Materials and Methods

### Patients and tissue samples

Placental tissue samples used in this study were randomly collected from 4 women with uncomplicated pregnancies and 4 women with pregnancies complicated by ICP in Wuxi Maternity and Child Health Care Hospital of Nanjing Medical University between May 2011 and September 2011. All subjects were primiparous Chinese women with a singleton pregnancy. ICP was diagnosed in women presenting with classical pruritus associated with liver dysfunction and raised serum bile acids, both of which resolved after delivery. All other causes of liver dysfunction, including preeclampsia, HELLP (hemolysis, elevated liver enzymes and low platelets) syndrome, acute fatty liver of pregnancy, primary biliary cirrhosis, viral hepatitis and any ultrasound abnormality that may result in biliary obstruction were excluded [9]. None of the patients received UDCA treatment. The characteristics of patients and the serum levels of bile acid are summarized in Table 1. The serum levels of bile acid were measured at diagnosis time. The placentas were collected from ICP patients and controls after cesarean section and the tissues were collected followed a previous published method [12]. Briefly, five different punches from various areas of each placenta were pooled randomly immediately after caesarian section, and washed in cold normal saline to eliminate any contaminating blood. The placental tissues were then immediately frozen in liquid nitrogen for proteomics research and western blotting. For immunohistochemistry and TUNEL experiments, placental tissues were fixed in PBS with 10% formalin for 24 h at 4°C. Then, they were dehydrated in a graded series of ethanol and embedded in paraffin. Ethical approval for this study was obtained from the Institutional Review Board of Nanjing Medical University and all participants signed an informed consent form.

**Table 1:** The clinical characteristics of pregnant women with ICP and healthy pregnant women.

Parameter	ICP	Control
Age (years)	29.5±4.65	28.7±2.38
Body weight	64.25±5.12	67±3.83
Gestational age (weeks)	36.36±1.17	38.11±0.88
TBA (µmol/L)	87.78±9.62*	3.33±1.27
ALT (IU/L)	178.98±97.62*	11.25±2.75
AST (IU/L)	138.32±52.25*	15.25±2.87

TBA, total bile acid; alanine transaminase, ALT; aspartate transaminase, AST; Statistical analysis was performed using *t*-tests; *P<0.05 was considered significant.

### Protein lysis, digestion and labeling with 8-plex iTRAQ reagents

To extract proteins from placental samples, frozen tissues were dissolved in a lysis buffer containing 7 M urea, 2 M thiourea, 2% (w/v) DTT and 1% (v/w) protease inhibitor cocktail at 4°C for 1 h, and then insoluble molecules were removed by centrifugation at 40,000 g for 1 h at 4°C. The supernatant was collected, and protein concentration was determined by the Bradford method using bovine serum albumin (BSA) as the standard as described previously [13]. Trypsin digestion and iTRAQ labeling were performed according to the manufacturer's protocol (Applied Biosystems, Foster City, CA, USA). Briefly, 100 µg protein from each sample was reduced, alkylated and then digested overnight at 37°C with trypsin (mass spectrometry grade; Promega, Madison, WI, USA) for 12 h, and labeled with iTRAQ reagents (Applied Biosystems) as follows: iTRAQ tag 113, control-1; 114, control-2; 115, control-3; 116, control-4; 117, patient-1; 118, patient-2; 119, patient-3 and 121, patient-4. The proteins were then mixed and dried using Labconco CentriVap system 79700 (Labconco Corporation, MO, USA) according to the manufacturer's protocol at 1500 rpm for about 3 h at 4°C.

### SCX fractionation

Labeled peptide mixtures were resuspended in SCX chromatography Buffer A (10 mM NH_4_COOH, 5% ACN, pH 2.7) and loaded onto a strong-cation exchange column (1 mm ID ×10 cm packed with Poros 10 S; DIONEX, Sunnyvale, CA, USA) for fractionation. Fractionation was performed using a linear salt gradient ammonium formate a flow-rate of 50 µl/min as follows: 0% to 30% B (800 mM NH_4_COOH, 5% ACN, pH 2.7) for 21 min; 30% to 56% B for 7 min; 56% to 100% B for 1 min; 100% B for 3 min; 100% to 0% B for 1 min; 0% B for 20 min before the next run, effluents were monitored at 214 nm based on the UV-light trace, and fractions were collected every 2 min; a total of 20 fractions were obtained.

### LC-MS/MS analysis

The twenty fractions were sequentially loaded onto a µ-precolumn™ cartridge (0.3×5 mm, 5 µm, 100 Å; DIONEX) at a flow rate of 20 µl/min. The trap column effluent was then transferred to a reverse-phase microcapillary column (0.075×150 mm, Acclaim® PepMap100 C18 column, 3 µm, 100 Å; DIONEX). Reverse-phase separation of peptides was performed using the following buffers: 2% ACN, 0.5% acetic acid (buffer A), and 80% ACN, 0.5% acetic acid (buffer B); a gradient was used (4% to 9% buffer B for 3 min, 9% to 33% buffer B for 170 min, 33% to 50% buffer B for 10 min, 50% to 100% buffer B for 1 min, 100% buffer B for 8 min, 100% to 4% buffer B for 1 min).

Peptide analysis was performed using LTQ Orbitrap Velos (ThermoFisher Scientific, San Jose, CA, USA) coupled directly to a LC column. An MS survey scan was obtained for the m/z range 350–1800, and ion trap collision-induced dissociation (CID)-MS/MS spectra were acquired from the survey scan for the 8 most intense ions, followed by higher-energy collision dissociation (HCD)-MS3 fragmentation of the most intense fragment ion in an m/z range between 400–800 of each of the 8 most intense ions (as determined by Xcalibur mass spectrometer software in real time). Dynamic mass exclusion windows of 60 s were used; siloxane (m/z 445.120025) was used as lock mass.

### Protein identification and quantification

Peak lists were generated using the software of the mass spectrometer. The raw files were used to search the International Protein Index (IPI) human proteome database (version 3.83; 93289 sequences) [14] using MaxQuant (version 1.2.2.5) [15]. A common contaminants database was also included for quality control. A reverse strategy was used to estimate the false discovery rate. Enzyme specificity was set as Trypsin/P (no Proline restriction); the maximum number of missed cleavage sites permitted was two. The minimum peptide length required was six. Carbamidomethylation of cysteine (+57 Da), and 8-Plex iTRAQ modification of N-term (+304 Da) and K (+304 Da) were set as fixed modifications, and oxidized methionine (+16 Da) was set as a variable modification. Mass tolerance for precursor ions and fragment ions were set to 20 ppm and 0.5 Da, respectively. The site, peptide and protein false discovery rates (FDR) were set to 0.01.

Protein quantification was calculated by combining the MaxQuant identification results with local Libra quantification algorithm [16], except that the quantification signals were extracted from the corresponding HCD-MS3 spectra. For the identification of differentially expressed proteins, the cutoffs for the fold change and *P* value (Student's t-test) were set to 1.5 and 0.05, respectively.

### Bioinformatic analysis

To obtain an overview of the expression levels of the differentially expressed proteins, 38 differentially expressed proteins from ICP and healthy pregnant women from iTRAQ data were analyzed by a hierarchical clustering method using MeV [17] (Table 2). An analysis of cellular processes influenced by differentially expressed proteins from ICP and healthy pregnant women was performed using PathwayStudio (v7.00) software (Ariadne Genomics, Inc. Rockville, MA) [18]. The cellular processes influenced by the differentially expressed proteins were determined by searching the database for the imported genes/proteins and for cellular processes in which the imported genes/proteins are involved. Each identified cellular process was confirmed manually using the relevant PubMed/Medline hyperlinked abstracts.

**Table 2:** Differentially expressed proteins in the placenta tissue from pregnant women with ICP and healthy pregnant women identified by iTRAQ labeling-based proteomics.

Accession no.	Gene name	Protein name	Fold change (P: C ratio)	*P* value	Expression change
IPI00000643	BAG2	BAG family molecular chaperone regulator 2	9.9	1.3E-02	Up
IPI00032328	KNG1	Isoform HMW of Kininogen-1	7.7	3.7E-02	Up
IPI00947127	LDHA	L-lactate dehydrogenase A chain isoform 3	3.7	4.9E-02	Up
IPI00968077	AFP	Alpha-fetoprotein	3.4	1.3E-02	Up
IPI00031479	PDIA5	Protein disulfide-isomerase A5	2.8	2.8E-02	Up
IPI00022229	APOB	Apolipoprotein B-100	2.8	3.3E-02	Up
IPI00022977	CKB	Creatine kinase B-type	2.7	2.1E-02	Up
IPI00917938	SERPINE2	glia-derived nexin isoform c precursor	2.5	8.7E-03	Up
IPI00216008	G6PD	Isoform Long of Glucose-6 -phosp-hate -dehydrogenase	2.5	2.8E-02	Up
IPI00007118	SERPINE1	Plasminogen activator inhibitor 1	2.5	1.6E-02	Up
IPI00945633	SSR1	Uncharacterized protein	2.2	4.6E-03	Up
IPI00024911	**ERp29**	Endoplasmic reticulum resident protein 29	2.0	1.2E-02	Up
IPI00479186	PKM2	Isoform M2 of Pyruvate kinase isozymes M1/M2	2.0	2.4E-02	Up
IPI00013475	TUBB2A	Tubulin beta-2A chain	1.9	3.0E-03	Up
IPI00465248	ENO1	Isoform alpha-enolase of Alpha-enolase	1.8	2.6E-02	Up
IPI00009904	PDIA4	Protein disulfide-isomerase A4	1.8	1.1E-02	Up
IPI00000105	MVP	Major vault protein	1.8	1.3E-02	Up
IPI00018335	FLT1	Isoform Flt1 of Vascular endothelial growth factor receptor 1	1.8	4.5E-02	Up
IPI00219018	GAPDH	Glyceraldehyde-3-phosphate dehydrogenase	1.8	2.4E-02	Up
IPI00023598	TUBB4	Tubulin beta-4 chain	1.7	4.3E-02	Up
IPI00220301	**PRDX6**	Peroxiredoxin-6	1.7	1.8E-02	Up
IPI00477729	ACOX1	Isoform 2 of Peroxisomal acylcoenzyme A oxidase 1	1.7	4.0E-02	Up
IPI00011229	CTSD	Cathepsin D	1.7	2.8E-02	Up
IPI00000816	YWHAE	Isoform 1 of 14-3-3 protein epsilon	1.7	4.6E-02	Up
IPI00217766	SCARB2	Lysosome membrane protein 2	1.6	4.2E-02	Up
IPI00031420	UGDH	UDP-glucose 6-dehydrogenase	1.6	4.6E-02	Up
IPI00216691	PFN1	Profilin-1	1.6	2.1E-02	Up
IPI00304866	TNFAIP2	Tumor necrosis factor alpha-induced protein 2	1.6	2.5E-02	Up
IPI00009923	P4HA1	Isoform 1 of Prolyl 4-hydroxylase subunit alpha-1	1.5	3.4E-02	Up
IPI00236556	**MPO**	Isoform H7 of Myeloperoxidase	3.5	5.9E-03	Down
IPI00456534	TTC7B	Isoform 2 of Tetratricopeptide repeat protein 7B	1.7	2.1E-02	Down
IPI00329482	LAMA4	Isoform 1 of Laminin subunit alpha-4	1.7	2.9E-02	Down
IPI00070943	PI4KA	Isoform 1 of Phosphatidylinositol 4-kinase alpha	1.7	1.5E-03	Down
IPI00790445	ANO6	anoctamin-6 isoform d	1.6	1.1E-02	Down
IPI00307017	LNPEP	Isoform 1 of Leucyl-cystinyl aminopeptidase	1.6	1.1E-02	Down
IPI00059279	EXOC4	Exocyst complex component 4	1.5	1.9E-03	Down
IPI00002406	BCAM	Basal cell adhesion molecule	1.5	4.7E-02	Down
IPI00026944	NID1	Isoform 1 of Nidogen-1	1.5	2.9E-02	Down

The table contains quantitative information for proteins which were at least > 1.5-fold upregulated or at least <0.67-fold downregulated in pregnant women with ICP (P) compared with healthy pregnant women (C), as defined in the experimental procedures. The key proteins verified by Western blot and immunohistochemisty analysis were highlighted in bold. The corresponding average ratios between the two groups (P:C) were given.

### Western blotting

Frozen tissues from 4 pregnant women with ICP and 4 healthy pregnant women were dissolved in a lysis buffer for protein extraction as described above, and samples containing 100 µg of protein from placental tissues of 4 ICP paitents and 4 normal controls were electrophoresed on a 12% SDS polyacrylamide gel and transferred to a nitrocellulose membrane (GE Healthcare, San Francisco, CA, USA). The membranes were blocked in Tris-buffered saline (TBS) containing 5% non-fat milk powder for 1 h, and incubated overnight with anti-ERp29 (ab42002, 1∶150; Abcam, Cambridge, MA, USA), anti-MPO (ab45977, 1∶500; Abcam), anti-PRDX6 (ab59543, 1∶500; Abcam) and anti-Tubulin (ab6046, 1∶1000; Abcam) antibodies diluted in TBS/5% non-fat milk powder. Tubulin was used as a loading control. The membranes were washed three times (10 min each) with TBS and incubated for 1 h with horseradish peroxidase (HRP)-conjugated goat anti-rabbit IgG (1∶1000; Beijing ZhongShan Biotechnology, Beijing, China). Specific proteins were detected using an ECL kit and AlphaImager (FluorChem5500; Alpha Innotech). The protein expression levels were analyzed using AlphaEaseFC software (Alpha Innotech, San Leandro, CA, USA).

### Immunohistochemistry

Formalin-fixed tissues from 4 pregnant women with ICP and 4 healthy pregnant women were embedded in paraffin, sectioned at 5 µm, and mounted on silane-coated slides. The sections were dewaxed and rehydrated through descending grades of alcohol to distilled water, followed by blocking of endogenous peroxidase using 3% (v/v) hydrogen peroxidase in phosphate buffered saline (PBS). The sections were subjected to microwave antigen retrieval in 0.02 M EDTA, washed in PBS and blocked with goat serum (Beijing ZhongShan Biotechnology) for 2 h, then incubated overnight at 4°C with anti-ERp29 (1∶200), anti-MPO (1∶200) and anti-PRDX6 (1∶200). Following three washes in PBS, the sections were incubated with HRP-conjugated secondary antibody (1∶1,000; Beijing ZhongShan Biotechnology) for 1 h at room temperature. Immunoreactivity was demonstrated using diaminobenzidine (Beijing ZhongShan Biotechnology) for increased sensitivity, which produced a brown insoluble precipitate at immunopositive sites. Sections were counterstained with hematoxylin and mounted with a cover glass. The negative controls were incubated with a solution that was devoid of any primary antibody. All immunostained sections were evaluated in a blinded manner by two observers.

### Assessment of placental apoptosis using the TUNEL assay

In order to investigate the appearance of apoptosis in the placental tissue from pregnant women with ICP, we quantified the apoptotic index in the placenta using TUNEL assay. Paraffin-embedded tissues were sectioned at 5 µm. Sections were then stained using the ApopTag in situ apoptosis detection kit (Millipore Corp, San Francisco, CA, USA) according to the manufacturer's instructions as described previously [19]. All the sections were evaluated in a blinded manner by two observers. For the assessment of apoptosis, ten high-power fields (×400 magnifications) were selected randomly in each specimen, and nuclear staining of trophoblastic cells was examined; the number of TUNEL-positive cells/field was counted to represent the apoptotic index [20].

### Statistical analysis

Statistical analysis was performed using SPSS software 16.0 (SPSS Inc., Chicago, IL, USA). The data was analyzed using Student's *t*-test and one way analysis of variance. The results were expressed as the mean ± SE. A *P*-value less than 0.05 was considered statistically significant.

## Results

### The clinical characteristics of pregnant women with ICP and healthy pregnant women

The clinical characteristics of 4 pregnant women with ICP and 4 healthy pregnant women were summarized in Table 1. There were no significant differences in the maternal ages, body weight between ICP group and normal control group (*P*>0.05). Although the delivery weeks of pregnant women with ICP were slightly earlier, there was no significant difference between pregnant women with ICP and healthy pregnant women (36.36±1.17 vs. 38.11±0.88, *P*>0.05). However, the levels of total bile acid (TBA), alanine transaminase (ALT) and aspartate transaminase (AST) in pregnant women with ICP were significantly higher than that in healthy pregnant women (87.78±9.62 µmol/L vs 3.33±1.27 µmol/L, 178.98 IU/L ±97.62 IU/L vs 11.25±2.75 IU/L and 138.32±52.25 IU/L vs 15.25±2.87 IU/L; *P*<0.05, respectively) (Table 1).

### Identification of differentially expressed proteins in the placental tissue from pregnant women with ICP and healthy pregnant women by iTRAQ Labeling and LC-MS/MS

**Figure 1:**
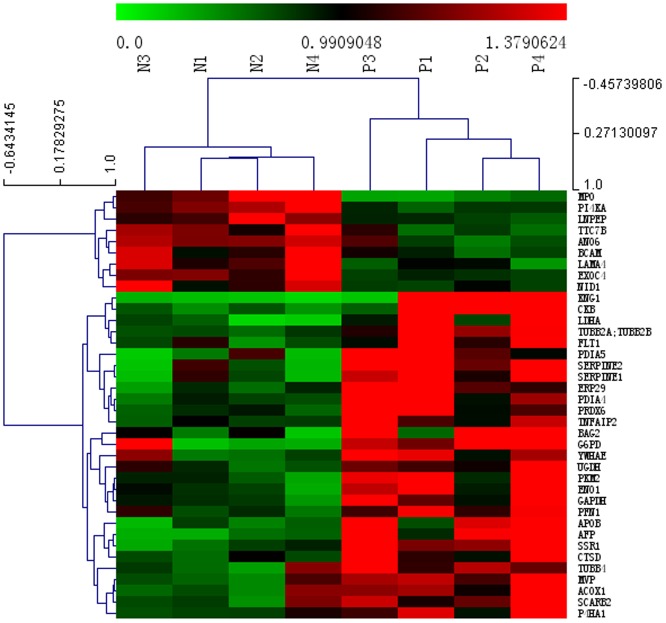
Cluster analysis of differentially expressed proteins in the placental tissue from pregnant women with ICP and healthy pregnant women. Hierarchical cluster analysis for the 38 differentially expressed proteins displaying significantly altered expression levels in the placental tissue from pregnant women with ICP and healthy pregnant women. “N” represents healthy pregnant women and “P” represents pregnant women with ICP. The protein expression levels are shown as colored boxes; red indicates a high expression level and green indicates a low expression level.

**Figure 2:**
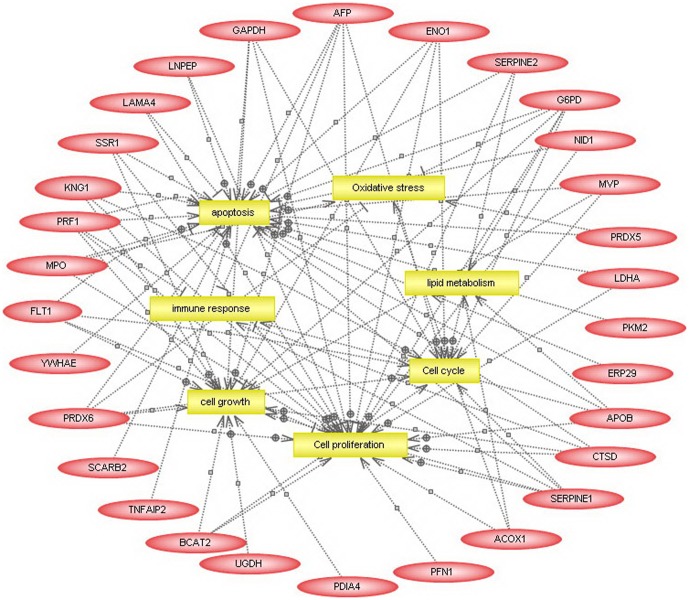
Bioinformatic analysis of differentially expressed proteins in the placental tissue from pregnant women with ICP and healthy pregnant women predicted by PathwayStudio™. Analysis of the cellular pathways affected by the differentially expressed proteins was performed using the PathwayStudio software. Proteins are shown as red ovals, regulated processes are represented by yellow squares. Regulation events are displayed with arrows and documented by literature citations.

2120 proteins (7399 peptides) were identified in the placental tissues from pregnant women with ICP and healthy pregnant women by iTRAQ and LC-MS/MS. 38 differentially expressed proteins were separated, of them 29 were upregulated and 9 were downregulated in placenta from pregnant women with ICP (Table 2). Cluster analysis was performed to characterize the specific and unique expression patterns of the 38 differentially expressed proteins, it displayed the significantly altered expression levels of the proteins in pregnant women with ICP and healthy pregnant women (Fig. 1). To gain a better understanding of the 38 proteins identified in this study, a detailed analysis of cellular processed influenced by these proteins was performed using PathwayStudio™ software. As shown in Fig. 2, most of the identified proteins were functionally related to specific cell processes, including apoptosis, oxidative stress, lipid metabolism, cell cycle, immune response, cell proliferation and cell growth. Of them, endoplasmic reticulum protein 29 (ERp29), peroxiredoxin 6 (PRDX6) and myeloperoxidase (MPO) are apoptosis related proteins.

### Western blot and immunohistochemical analysis of protein identity

**Figure 3:**
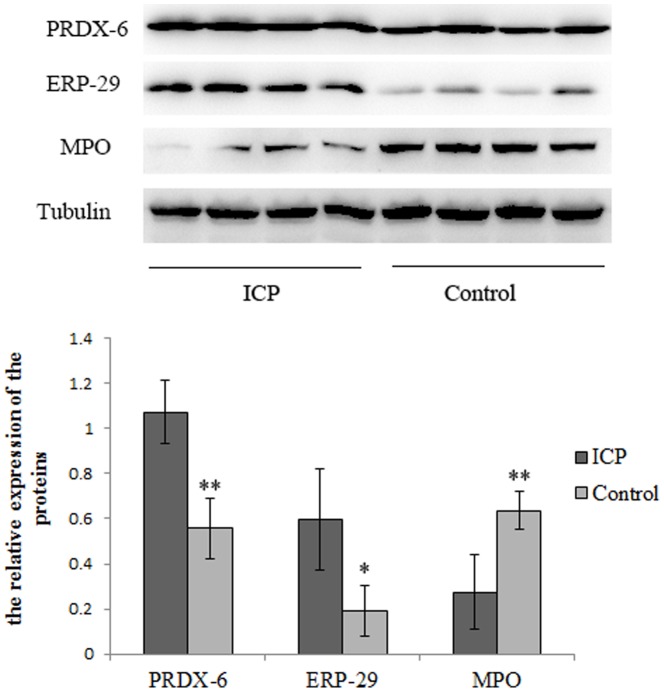
Western blot analysis of PRDX6, ERp29 and MPO in the placental tissue from pregnant women with ICP and healthy pregnant women. The expression levels of PRDX6 and ERp29 were significantly upregulated, whereas MPO was significantly downregulated in placenta from pregnant women with ICP compared to that in placenta from healthy pregnant women (*P* = 0.002, *P* = 0.018 and *P* = 0.009, respectively); β-tubulin was used as an internal control. (*P<0.05; **P<0.01).

**Figure 4:**
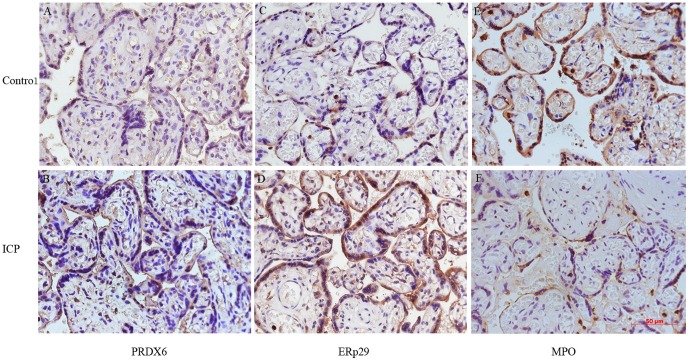
Immunohistochemical staining for PRDX6, ERp29 and MPO in the placental tissue from pregnant women with ICP and healthy pregnant women (×400). Immunohistochemstry images demonstrated higher expression of PRDX6 (B) and ERp29 (D), and lower expression of MPO (F) in cytoplasm and/or nucleus of trophoblasts in the placenta from pregnant women with ICP than those in placenta from healthy pregnant women (A, C, E).

Western blot analysis and immunohistochemistry were performed to validate the proteomic findings. ERp29, PRDX6 and MPO are apoptosis related proteins and have not been investigated in the pathogenesis of ICP, we then further verified the roles of ERp29, PDRX6 and MPO in the pathogenesis of ICP. The expression levels of ERp29 and PRDX6 were significantly increased, and the expression level of MPO was significantly reduced in the placenta from pregnant women with ICP compared to that in the placenta from healthy pregnant women measured by western blot (Fig. 3, *P*<0.05; respectively). We also performed immunohistochemical analysis to define the cellular location of these proteins in human placenta. As shown in Fig. 4, all three proteins were expressed predominantly in the cytoplasm and/or nucleus of the syncytiotrophoblastic and/or cytotrophoblastic cells of human placental tissue. The expression patterns of the three proteins were consistent with the MS and Western blot analysis.

### Apoptosis was increased in the placenta from pregnant women with ICP

**Figure 5:**
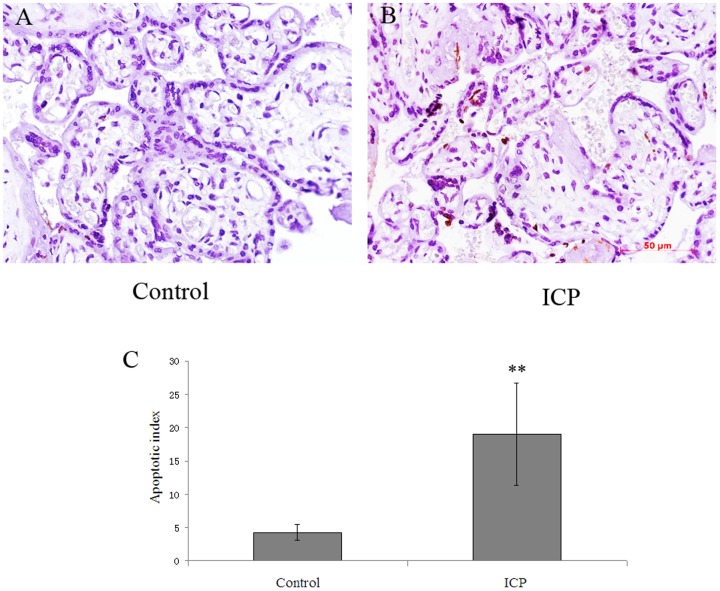
Comparison of the incidence of apoptosis in placenta from pregnant women with ICP compared to that in placenta from healthy pregnant women (×400). TUNEL assay demonstrated the incidence of apoptosis in placenta from pregnant women with ICP was higher than that in placenta from healthy pregnant women (A and B). Semi-quantitative analysis indicated there was significantly higher incidence of apoptosis in placenta from pregnant women with ICP (*P* = 0.007) (C).

As above our above proteomic results showed the expression of apoptosis related proteins were changed, we then further investigated the appearance of apoptosis in the placenta from pregnant women with ICP. As shown in Fig. 5, there were few TUNEL-positive cells in the placental tissue from healthy pregnant women (A), whereas TUNEL-positive cells in trophoblasts were significantly increased in the placental tissue from pregnant women with ICP (B) quantified by TUNEL assay.

## Discussion

To better understanding the pathogenesis of ICP, an iTRAQ-based proteomics approach was performed in this study. Our findings suggested that a total of 38 differentially expressed proteins were identified, of them 29 were upregulated and 9 were downregulated in placenta from pregnant women with ICP. The hierarchical cluster analysis also revealed significantly altered protein expression patterns between pregnant women with ICP and healthy pregnant women. Bioinformatic analysis revealed that most of the 38 proteins were functionally related to specific cell processes, including apoptosis, oxidative stress, lipid metabolism, cell cycle, immune response, cell proliferation and cell growth. Jing et al. [21] have identified differentially expressed genes in the placental tissues of ICP patients compared with normal controls, and most of the differentially expressed genes were involved in apoptosis, cell growth, immune response and transportation, which were almost consisted with our LC-MS/MS data. To the best of our knowledge, this is the first report of a proteomics-wide analysis of protein expression in placenta from pregnant women with ICP. Our findings provided the evidences that placental apoptosis, growth dysfunction, as well as immune maladaptation and disordered lipid metabolism were important pathophysiology of ICP and were possibly participated in the pathogenesis of ICP.

To validate the proteomic result and further investigate the preliminary function of the identified proteins, based on the availability of antibodies three apoptosis related proteins, ERp29, MPO and PRDX6 which have not been investigated in the placenta from pregnant women with ICP were chosen for further analysis. The expression patterns of the three proteins were analyzed by Western blot and immunohistochemical analysis, and the results confirmed that PRDX6 and ERp29 were upregulated, whereas MPO was downregulated in ICP, compared to controls, which confirming the LC-MS/MS data (*P*<0.05, respectively; Fig. 3 and Fig. 4). We also demonstrated increased apoptosis in the placental tissue of ICP patients (Fig. 5), which may be the primary pathological event and result in damage to the trophoblast structure and function of placenta causing reduced oxygen and nutrient transfer to the fetus and finally result in the incidence of adverse fetal outcomes. However, the mechanism of apoptosis was not clearly indentified.

Recently, the protein ERp29 has gained much research interest. ERp29 is a recently identified endoplasmic reticulum protein which is associated with the induction of apoptosis and oxidative stress [22]. Studies have demonstrated that ERp29 can activate the pathways which mediate caspase activation and apoptosis [23]. Overexpression of ERp29 may result in G0/G1 arrest and inhibit breast cancer cell proliferation [24]. Using proteomic analysis, Fang et al. identified that elevated ERp29 expression may be responsible for the apoptosis induced by curcumin in MCF-7 breast cancer cells [25]. Moreover, increased expression of ERp29 was detected in the placenta from women with preeclampsia using proteomics analysis and it may relate to the oxidative stress and apoptosis [26]. In this study, we also observed significantly increased ERp29 expression in the placenta of ICP patients, which suggested that overexpression of ERp29 may play a key role and participate in the induction of apoptosis in the placental tissue of ICP patients.

PRDX6 is a novel peroxidase enzyme, and an anti-oxidant protein. Oxidative stress can induce PRDX6 expression [27]. Overexpression of PRDX6 protects cells against oxidative damage and inhibits apoptosis in lung epithelial cells [28]. Downregulation of PRDX6 in a lung epithelial cell line resulted in increased sensitivity to oxidants and apoptosis [29]. In our study, overexpression of PRDX6 was observed in the placental tissue of ICP patients. We speculate that overexpression of PRDX6 may be a compensatory response in human body, which may play an anti-oxidant role and protect the placenta against oxidative damage and apoptosis in ICP patient. MPO is a peroxidase enzyme and abundantly expressed in neutrophil granulocytes. It functions to prevent apoptosis through inhibition of caspase-3 activation [30]. Overexpression of MPO has been detected in ovarian cancer, and silencing of MPO gene expression induced apoptosis in cancer cells through activation of caspase-3 [31]. Our observation that MPO is downregulated in ICP patients provides evidence that a reduction in the anti-apoptotic effect of MPO is associated with increased apoptosis in the placenta of ICP patients.

High concentrations of bile acids can induce several morphological abnormalities including increased number of syncytial knots [9], [32], and increased expression of apoptotic markers in the placental tissues in vivo and in vitro [1], [9]. It should be noted that although the vivo study in rodent model with cholestasis was much more acute and severe than is usually seen in patients with ICP, the findings is meaningful for investigation of the pathophysiology of ICP. Syncytial knots are thought to be sequestrations of degenerating nuclei and proposed to represent areas of increased apoptosis. The number of syncytial knots is increased in several pregnancy complications including pre-eclampsia, diabates and ICP, which were associated with increased placental apoptosis [9], [32]. Studies also reported that the syncytial knot formation was the effects of bile acids on placental morphology in ICP [9], [32], and apoptosis played a key role in the bile acid-induced morphological abnormalities [9]. All these findings suggested that placental apoptosis induced by high concentrations of bile acids is one of the mechanisms response to development of ICP and the pathophysiology of fetal complications. In this study our data showed that the expression of ERp29 and PRDX6 levels were increased, and the expression level of MPO was reduced in placenta from pregnant women with ICP. Our data also showed TUNEL-positive trophoblast cells in placenta from pregnant women with ICP were significantly increased, suggesting ERp29, PRDX6 and MPO are involved in the apoptosis in ICP.

In conclusion, our preliminary data using an iTRAQ-based proteomics approach indicates that ERp29, MPO and PRDX6 might play key roles in placental apoptosis in ICP patients. These results might provide us some new insights into the pathophysiology and potential novel treatment targets for ICP. However, our study is a preliminary work with the limitation that the data was based on a small number of samples. So, we will perform further study with expanded cohort in future, which will contribute to shedding light on the target treatment of ICP.

## References

[1] Perez MJ, Macias RI, Marin JJ (2006) Maternal cholestasis induces placental oxidative stress and apoptosis. Protective effect of ursodeoxycholic acid. Placenta 27: 34–41. 16310035 10.1016/j.placenta.2004.10.020

[2] Rook M, Vargas J, Caughey A, Bacchetti P, Rosenthal P, et al (2012) Fetal outcomes in pregnancies complicated by intrahepatic cholestasis of pregnancy in a Northern California cohort. PLoS One 7: e28343. 22403605 10.1371/journal.pone.0028343 PMC3293870

[3] Glantz A, Reilly SJ, Benthin L, Lammert F, Mattsson LA, et al (2008) Intrahepatic cholestasis of pregnancy: Amelioration of pruritus by UDCA is associated with decreased progesterone disulphates in urine. Hepatology 47: 544–51. 17968976 10.1002/hep.21987

[4] Lee RH, Incerpi MH, Miller DA, Pathak B, Goodwin TM (2009) Sudden fetal death in a) intrahepatic cholestasis of pregnancy. Obstet Gynecol 113: 528–31. 19155945 10.1097/AOG.0b013e31818db1c9

[5] Dixon PH, van Mil SW, Chambers J, Strautnieks S, Thompson RJ, et al (2009) Contribution of variant alleles of ABCB11 to susceptibility to intrahepatic cholestasis of pregnancy. Gut 58: 537–44. 18987030 10.1136/gut.2008.159541

[6] Glantz A, Marschall HU, Mattsson LA (2004) Intrahepatic cholestasis of pregnancy: relationships between bile acid levels and fetal complication rates. Hepatology 40: 467–4. 15368452 10.1002/hep.20336

[7] Marin JJG, Macias RIR, Serrano MA (2003) The hepatobiliary-like excretory function of the placenta. A review. Placenta 24: 431–8. 12774824 10.1053/plac.2002.0951

[8] Marin JJG, Briz O, Serrano MA (2004) A review on the molecular mechanisms involved in the placental barrier for drugs. Curr Drug Deliv 1: 275–89. 16305390 10.2174/1567201043334731

[9] Geenes VL, Lim YH, Bowman N, Tailor H, Dixon PH, et al (2011) A placental phenotype for intrahepatic cholestasis of pregnancy. Placenta 32: 1026–32. 22015023 10.1016/j.placenta.2011.09.006

[10] Geenes V, Williamson C (2009) Intrahepatic cholestasis of pregnancy. World J Gastroenterol 15: 2049–66. 19418576 10.3748/wjg.15.2049 PMC2678574

[11] Bacq Y, Sentilhes L, Reyes HB, Glantz A, Kondrackiene J, et al (2012) Efficacy of Ursodeoxycholic Acid in Treating Intrahepatic Cholestasis of Pregnancy: A Meta-analysis. Gastroenterology 143: 1492–501. 22892336 10.1053/j.gastro.2012.08.004

[12] Gharesi-Fard B, Zolghadri J, Kamali-Sarvestani E (2010) Proteome differences of placenta between pre-eclampsia and normal pregnancy. Placenta 31: 121–5. 19954843 10.1016/j.placenta.2009.11.004

[13] Zhao C, Guo XJ, Shi ZH, Wang FQ, Huang XY, et al (2009) Role of translation by mitochondrial-type ribosomes during sperm capacitation: an analysis based on a proteomic approach. Proteomics 9: 1385–99. 19253287 10.1002/pmic.200800353

[14] Kersey PJ, Duarte J, Williams A, Karavidopoulou Y, Birney E, et al (2004) The International Protein Index: an integrated database for proteomics experiments. Proteomics 4: 1985–8. 15221759 10.1002/pmic.200300721

[15] Cox J, Mann M (2008) MaxQuant enables high peptide identification rates, individualized p.p.b.-range mass accuracies and proteome-wide protein quantification. Nat Biotechnol 26: 1367–72. 19029910 10.1038/nbt.1511

[16] Deutsch EW, Mendoza L, Shteynberg D, Farrah T, Lam H, et al (2010) A guided tour of the Trans-Proteomic Pipeline. Proteomics 10: 1150–9. 20101611 10.1002/pmic.200900375 PMC3017125

[17] Saeed AI, Bhagabati NK, Braisted JC, Liang W, Sharov V, et al (2006) TM4 microarray software suite. Methods Enzymol 411: 134–93. 16939790 10.1016/S0076-6879(06)11009-5

[18] Nikitin A, Egorov S, Daraselia N, Mazo I (2003) Pathway studio-the analysis and navigation of molecular networks. Bioinformatics 9: 2155–7. 10.1093/bioinformatics/btg290 14594725

[19] Belkacemi L, Chen CH, Ross MG, Desai M (2009) Increased placental apoptosis in maternal food restricted gestations: role of the Fas pathway. Placenta 30: 739–51. 19616844 10.1016/j.placenta.2009.06.003

[20] Lee H, Park H, Kim YJ, Kim HJ, Ahn YM, et al (2005) Expression of lectin-like oxidized low-density lipoprotein receptor-1 (LOX-1) in human preeclamptic placenta: possible implications in the process of trophoblast apoptosis. Placenta 26: 226–33. 15708124 10.1016/j.placenta.2004.05.012

[21] Wei J, Wang H, Yang X, Dong M, Wang Z (2010) Altered gene profile of placenta from women with intrahepatic cholestasis of pregnancy. Arch Gynecol Obstet 281: 801–10. 19565256 10.1007/s00404-009-1156-3

[22] Zhang D, Richardson DR (2011) Endoplasmic reticulum protein 29 (ERp29): An emerging role in cancer. Int J Biochem Cell Biol 43: 33–6. 20920593 10.1016/j.biocel.2010.09.019

[23] Rao RV, Ellerby HM, Bredesen DE (2004) Coupling endoplasmic reticulum stress to the cell death program. Cell Death Differ 11: 372–80. 14765132 10.1038/sj.cdd.4401378

[24] Bambang IF, Xu S, Zhou J, Salto-Tellez M, Sethi SK, et al (2009) Overexpression of endoplasmic reticulum protein 29 regulates mesenchymal-epithelial transition and suppresses xenograft tumor growth of invasive breast cancer cells. Lab Invest 89: 1229–42. 19770839 10.1038/labinvest.2009.87

[25] Fang HY, Chen SB, Guo DJ, Pan SY, Yu ZL (2011) Proteomic identification of differentially expressed proteins in curcumin-treated MCF-7 cells. Phytomedicine 18: 697–703. 21239154 10.1016/j.phymed.2010.11.012

[26] Kim YN, Kim HK, Warda M, Kim N, Park WS, et al (2007) Toward a better understanding of preeclampsia: Comparative proteomic analysis of preeclamptic placentas. Proteomics Clin Appl 1: 1625–36. 21136660 10.1002/prca.200700034

[27] Chowdhury I, Mo Y, Gao L, Kazi A, Fisher AB, et al (2009) Oxidant stress stimulates expression of the human peroxiredoxin 6 gene by a transcriptional mechanism involving an antioxidant response element. Free Radic Biol Med 46: 146–53. 18973804 10.1016/reeradbiomed.2008.09.027 PMC2646855

[28] Manevich Y, Sweitzer T, Pak JH, Feinstein SI, Muzykantov V, et al (2002) 1-Cys peroxiredoxin overexpression protects cells against phospholipid peroxidation -mediated membrane damage. Proc Natl Acad Sci USA 99: 11599–604. 12193653 10.1073/pnas.182384499 PMC129315

[29] Pak JH, Manevich Y, Kim HS, Feinstein SI, Fisher AB (2002) An antisense oligonucleotide to 1-cys peroxiredoxin causes lipid peroxidation and apoptosis in lung epithelial cells. J Biol Chem 277: 49927–34. 12372839 10.1074/jbc.M204222200

[30] El Kebir D, József L, Pan W, Filep JG (2008) Myeloperoxidase delays neutrophil apoptosis through CD11b/CD18 integrins and prolongs inflammation. Circ Res 103: 352–9. 18617697 10.1161/01.RES.0000326772.76822.7a

[31] Saed GM, Ali-Fehmi R, Jiang ZL, Fletcher NM, Diamond MP, et al (2010) Myeloperoxidase serves as a redox switch that regulates apoptosis in epithelial ovarian cancer. Gynecol Oncol 116: 276–81. 19962178 10.1016/j.ygyno.2009.11.004 PMC2834266

[32] Costoya AL, Leontic EA, Rosenberg HG, Delgado MA (1980) Morphological study of placental terminal villi in intrahepatic cholestasis of pregnancy: histochemistry, light and electron microscopy. Placenta 1: 361–8. 7454694 10.1016/s0143-4004(80)80038-5

